# Serum CXCL13 levels are associated with lymphoma risk and lymphoma occurrence in primary Sjögren’s syndrome

**DOI:** 10.1007/s00296-020-04524-5

**Published:** 2020-02-11

**Authors:** Emmanuella Young Traianos, James Locke, Dennis Lendrem, Simon Bowman, Ben Hargreaves, Victoria Macrae, UK primary Sjögren’s syndrome registry, Jessica Rachael Tarn, Wan-Fai Ng

**Affiliations:** 1 Musculoskeletal Research Group, Faculty of Medical Sciences, Institute of Cellular Medicine, Newcastle University, Framlington Place, Newcastle upon Tyne, NE2 4HH UK; 2 Univesity Hospital Birmingham, Birmingham, UK; 3 NIHR Newcastle Biomedical Research Center, Newcastle upon Tyne, UK

**Keywords:** CXCL13, Germinal centres, non-Hodgkin lymphoma, Primary Sjögren’s syndrome

## Abstract

Primary Sjögren’s syndrome (pSS) is an autoimmune disease characterised by an increased risk for non-Hodgkin lymphoma (NHL) development. Ectopic germinal centre (GC) in the salivary gland is associated with increased NHL risk in pSS, and the chemokine CXCL13 is implicated in B-cell migration and GC formation. Serum CXCL13 concentrations were quantified by ELISA in 48 healthy individuals, 273 pSS patients without NHL (pSS-nonL), and 38 pSS patients with NHL (pSS-NHL+) from the United Kingdom Primary Sjögren’s Syndrome Registry cohort. PSS-nonL patients were stratified into low risk (LR), moderate risk (MR) and high risk (HR) groups according to the lymphoma risk score proposed by Fragkioudaki et al. Differences in serum CXCL13 levels among groups were analysed using the Wilcoxon method. Also, changes in serum CXCL13 over a time period of at least 1 year and a median 4 years were assessed for 200 pSS-nonL and 8 pSS-NHL+ patients. In addition, associations of serum CXCL13 with B-cell and inflammatory markers were investigated by correlation analyses and logistic regression. Serum CXCL13 levels were higher in all pSS groups compared to controls (*p* < 0.0001), and in pSS-NHL+ compared to pSS-nonL patients (*p* = 0.0204). LR patients had lower CXCL13 levels than MR patients (*p* < 0.0001) and pSS-NHL+ patients (*p* = 0.0008). CXCL13 levels remained stable over the study period for all pSS groups. CXCL13 was associated (*p* < 0.0005) with Immunoglobulin G (IgG), B-cell activating factor, β2 microglobulin, combined free light chains, κ and λ light chains, anti-Ro/SSA, anti-La/SSB, and erythrocyte sedimentation rate. IgG and C3 controlled for age and gender were significantly associated with NHL risk in pSS. Serum CXCL13 levels were elevated in pSS-NHL+ and MR patients compared to LR patients and remained stable over time. Further study is required to investigate the role of CXCL13 in pSS-associated NHL risk.

**Electronic supplementary material:**

The online version of this article (10.1007/s00296-020-04524-5) contains supplementary material, which is available to authorized users.

## Introduction

Primary Sjögren’s syndrome (pSS) is a chronic autoimmune disease with a wide clinical spectrum that extends from lacrimal and salivary gland (SG) exocrinopathy to systemic manifestations with an increased risk for B-cell NHL [1–3]. NHL is a serious complication in pSS affecting around 5–10% of patients. The relative increased risk compared to the general population is estimated to be 9- to 17-fold [1–4]. Lymphoma pathogenesis in pSS is a multifactorial process in which chronic antigenic stimulation in genetically predisposed individuals with pSS may trigger polyclonal and later oligoclonal B-cell activation leading to the development of malignant B-cell NHL [5].

One of the most frequently reported histopathological characteristic indicatives of high risk for pSS-associated NHL is the formation of ectopic GC in the SG epithelium [6]. Ectopic GC formation is regulated by cytokines and chemokines such as B-cell activating factor (BAFF), which mediates T-cell activation and B-cell survival, and has been associated with lymphomagenesis [7–9].

The chemokine (C-X-C motif) ligand 13 (CXCL13) and its CXC chemokine receptor 5 (CXCR5) play a critical role in the maintenance of ectopic tertiary lymphoid structures, the organisation of B-cell follicles and the migration of B cells into ectopic GC [10, 11]. CXCL13, also known as B-cell chemoattractant (BCA-1), is produced by follicular dendritic cells and GC T follicular helper cells, in secondary lymphoid organs [12, 13]. The important role of the CXCL13–CXCR5 pair in vivo has been shown by the impaired migration of activated B cells into splenic follicles in CXCR5 knockout mice and their failure to form lymph nodes [14, 15]. Moreover, using transgenic mice expressing only CXCR5 in β cells of the pancreatic islets demonstrated that CXCL13 expression was sufficient not only to mediate B-cell recruitment but also to induce lymphoid neogenesis [16]. Furthermore, the development of a two-dimensional model showed that CXCL13/CXCR5 signalling enhances antigen encounter with BCR-triggered B-cell activation by shaping cell dynamics [17].

CXCL13 is found in human blood, plasma and serum and high CXCL13 levels have been associated with pSS and systemic lupus erythematosus (SLE) [18–21]. A previous study that included patients from the Assessment of Systemic Signs and Evolution of Sjögren’s Syndrome (ASSESS) cohort showed that serum CXCL13 and CCL11 levels were elevated in pSS patients with high EULAR Sjögren’s Syndrome Disease Activity Index (ESSDAI) score, B-cell activation and NHL [19].

The aim of the current study was to investigate the potential of CXCL13 to assess NHL risk in pSS. The establishment of additional easily measured NHL risk biomarkers can contribute to improved patient management and stratification of pSS patients.

## Materials and methods

### Study participants

Study participants were selected from the United Kingdom Primary Sjögren’s Syndrome Registry (UKPSSR) cohort which includes approximately 700 pSS patients and 400 healthy controls who do not have an autoimmune condition [22]. All pSS patients fulfilled the American-European Consensus Classification Criteria (AECG) for pSS diagnosis and all study participants provided written informed consent to be included in the registry. This study has included the following subject groups: (1) 48 healthy individuals, which was considered a sufficient number of controls for this study, (2) 273 pSS-nonL patients [200 had follow-up (visit 2) cases], and (3) 38 pSS-NHL+ patients (8 had visit 2 cases). The minimum follow-up time limit for the patients was 1 year and the median time, 4 years. Demographics and disease characteristics of the study population are shown in Table 1. All clinical data were collected contemporaneously at the time of recruitment.

**Table 1:** Demographics and disease characteristics of primary Sjögren’s syndrome patients without and with non-Hodgkin lymphoma at visit 1 and visit 2

	pSS patients without NHL		pSS patients with NHL	
	Visit 1	Visit 2	Visit 1	Visit 2
No. of pSS patients	273	200	38	8
Female gender (%)	91	90	95	81
Age, years median (Q1, Q3)	64 (54, 70)	65 (55, 71)	59 (42.8, 68.3)	48.5 (44, 73)
pSS duration from AECG diagnosis, years median (Q1, Q3)	4 (1, 9)	8 (5, 15)	7.5 (2, 14.3)	9.5 (5.3, 17)
pSS duration from symptoms onset, years median (Q1, Q3)	10 (5, 16)	15 (8, 21.8)	15.5 (7, 27.5)	15.5 (10, 30.8)
ESSDAI score median (Q1, Q3)	4 (1, 8)	2 (0, 7)	6 (2, 13)	1 (0, 13)
ESSPRI score median (Q1, Q3)	5.6 (4, 7)	6 (5, 7.7)	5.6 (4, 7.6)	6 (3.6, 7.8)

*AECG* American-European consensus classification criteria, *ESSDAI* EULAR Sjögren’s syndrome disease activity index, *ESSPRI* EULAR Sjögren’s syndrome patient reported index

### Patient stratification into NHL risk groups

PSS-nonL patients were categorised into low, moderate and high NHL risk groups using the risk assessment tool proposed by Fragkioudaki et al. [23]. PSS-NHL+ patients consisted of 38 patients with a history of NHL (Table S1, Supplementary Material 1). The NHL risk score was calculated based on the number of independent risk factors at pSS diagnosis according to Fragkioudaki’s risk assessment tool for NHL development [23]. The NHL risk factors were persistent SG enlargement, lymphadenopathy, Raynaud’s phenomenon, Ro/SSA and/or La/SSB autoantibodies, rheumatoid factor (RF) positivity, monoclonal gammopathy, and C4 hypocomplementemia. Patients with ≤ 2 risk factors are considered low risk (LR), patients with between 3 and 6 risk factors are considered moderate risk (MR) and patients having all 7 risk factors are considered high risk (HR). In our study, there were 129 LR, 143 MR, 38 pSS-NHL+ and only 1 HR patients. This patient developed lymphoma 6 years after visit 1.

### Measurement of serum CXCL13 levels

Peripheral blood samples were obtained at visit 1 and visit 2, and serum was extracted and stored at − 80 °C until use. Serum CXCL13 concentrations were quantified using the BLC/CXCL13 human enzyme-linked immunosorbent assay (ELISA) kit according to the manufacturer’s instructions (ThermoFisher Scientific).

### Measurement of serum B-cell and inflammatory markers

Anti-Ro/SSA, anti-La/SSB, RF, IgG, immunoglobulin A (IgA), immunoglobulin M (IgM), C3, C4, ESR, white blood cell count (WCC) and C-reactive protein (CRP) were measured by the clinical laboratory of the recruiting hospitals. BAFF, β2 microglobulin (B2M), κ light chain, λ light chain, κ–λ ratio, and combined free light chains (CMBY) were measured by The Binding Site (Birmingham, UK) as previously described [24].

### Statistical analysis

Visual checks of the distribution of variables were made and log transformation was performed for positively skewed data. Differences in serum CXCL13 concentrations between risk groups, and differences in CXCL13 between visit 1 and visit 2 patients were analysed using the Kruskal–Wallis test and Wilcoxon signed-rank test, respectively. Correlation analyses were performed to find associations of CXCL13 with B-cell and inflammatory markers, and were expressed as Pearson correlation coefficients (r). Logistic regression analysis was performed to model NHL risk against CXCL13, and other candidate biomarkers. Data are presented as medians and interquartile ranges (IQR) for continuous variables and as numbers or percentages for categorical variables. All statistical tests were performed in JMP statistical software (Version 13).

## Results

### Serum CXCL13 levels are associated with pSS and pSS-associated NHL risk and occurrence

**Fig. 1:**
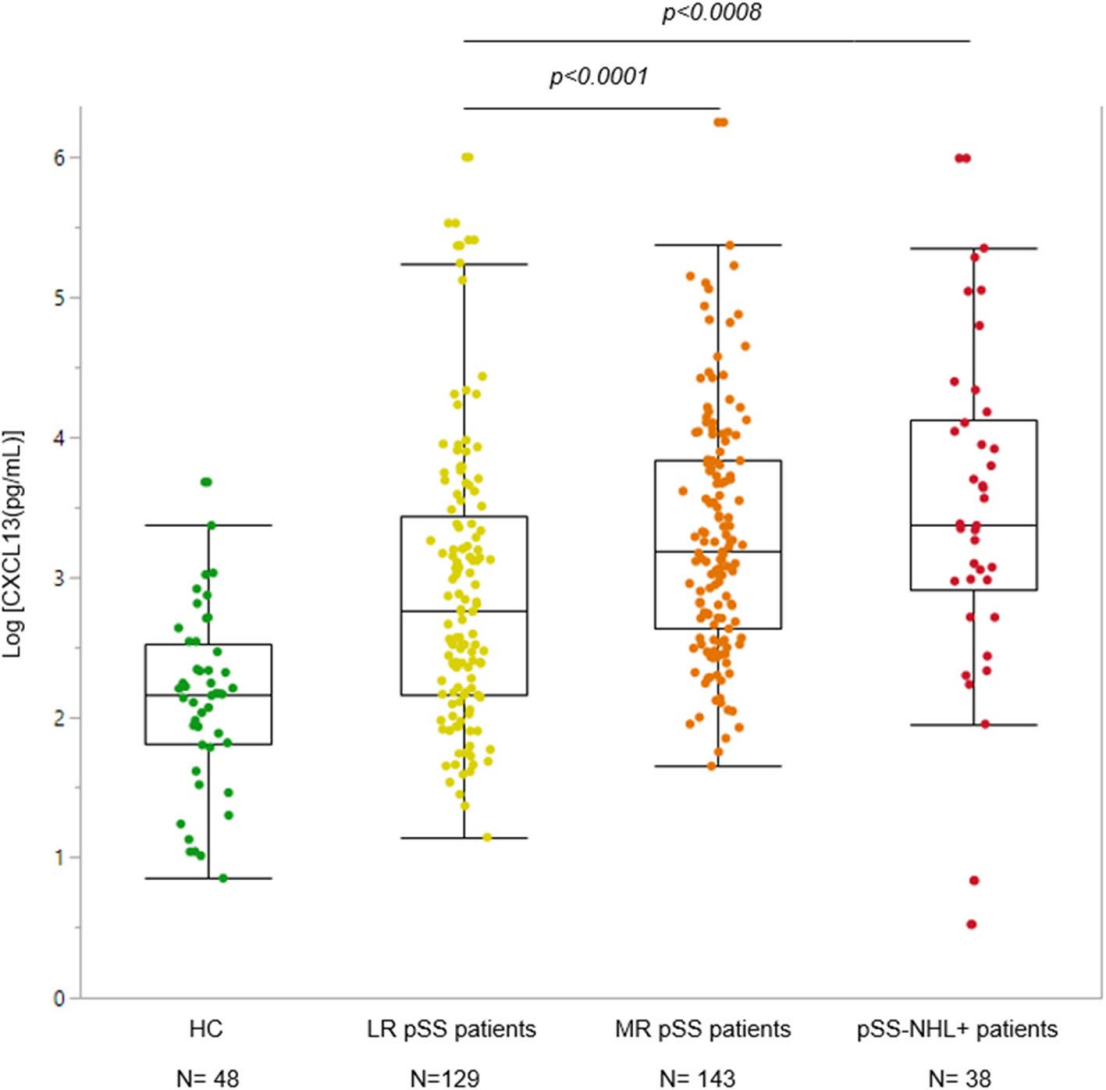
Comparison of serum CXCL13 levels among healthy controls (HC), low risk (LR) pSS, moderate risk (MR) pSS and pSS with non-Hodgkin lymphoma (pSS-NHL+) patients at visit 1. Serum CXCL13 levels were higher in all pSS patients when compared with HC (*p* < 0.0001). CXCL13 levels were lower in the low risk group when compared with the moderate risk (*p* < 0.0001) and the pSS-NHL+ group (*p* = 0.0008) but not significantly different between the pSS-NHL+ and the moderate risk group. Comparisons of CXCL13 concentrations among groups were performed using the Kruskal–Wallis test. The boxes show the median CXCL13 concentration and the quartiles Q1 and Q3. Each dot represents an individual patient. The values beyond the whiskers are outliers. The analysis included all values including these outliers

Serum concentrations of CXCL13 were significantly elevated in all pSS groups compared to healthy controls (*p* < 0.0001) (see Fig. 1) and in pSS-NHL+ patients compared to pSS-nonL patients (*p* = 0.0204) (see Supplementary Fig. S2). At visit 1, LR patients had lower serum CXCL13 levels than MR patients (*p* < 0.0001) and pSS-NHL+ patients (*p* = 0.0008). No significant difference was found between MR and pSS-NHL+ patients (see Fig. 1).

### Serum CXCL13 levels and NHL risk score remained stable between the two visits

**Fig. 2:**
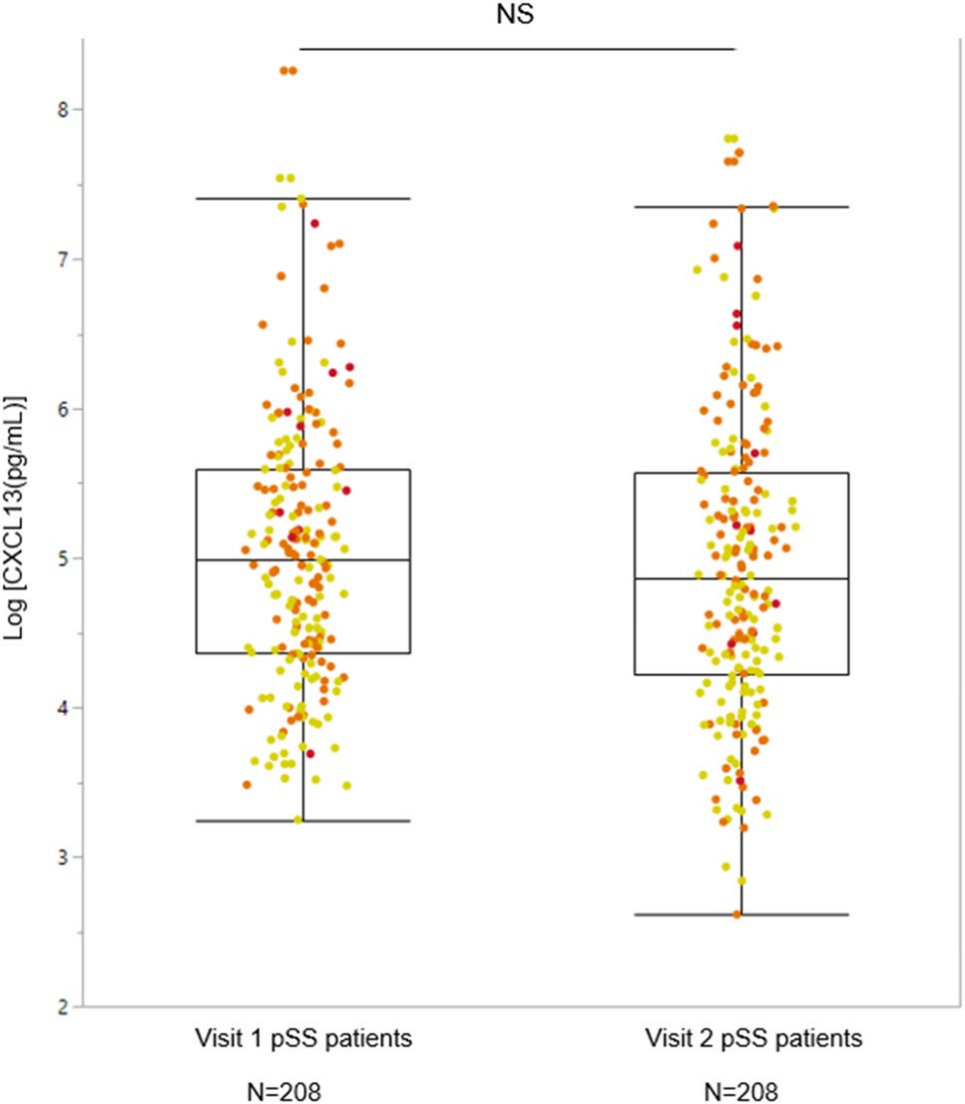
Comparison of serum CXCL13 levels between visit 1 and visit 2 patients with pSS using the Wilcoxon Signed Rank test. No significant difference was found in the serum CXCL13 concentrations between visit 1 and visit 2 patients. Number of matched paired pSS patients: 208. The boxes show the median CXCL13 concentration the quartiles Q1 and Q3. Each dot represents an individual patient. The values beyond the bars are outliers that were included in the analysis. Yellow dot colour: Low risk pSS patients, Orange dot colour: Moderate risk pSS patients. Red dot colour: pSS patients with NHL

We then compared the CXCL13 concentrations in 208 matched paired patient samples with visit 1 and visit 2. We found that serum CXCL13 levels remained stable between the two visits (see Fig. 2). NHL risk score remained unchanged between the two time points. In the LR group, 99% patients remained in the same group, 1% changed to the MR group and none developed NHL. In the MR group, 92% patients remained in the same group, 5.4% changed to the LR group and 2.2% developed NHL.

### CXCL13 is associated with B-cell and inflammatory markers

Correlation analyses were performed investigating the relationship between CXCL13 and other B-cell and inflammatory markers. There were statistically significant associations with BAFF (*r* = 0.263, *p* = 0.0002), B2M (*r* = 0.459, *p* < 0.0001), CMBY (*r* = 0.375, *p* < 0.0001), κ light chain (*r* = 0.358, *p* < 0.0001), λ light chain (*r* = 0.338, *p* < 0.0001), IgG (*r* = 0.320, *p* < 0.0001), and RF (*p* = 0.0039) (see Table 2). In addition, there was a statistically significant difference in Ro/La status: patients with anti-Ro/SSA and anti-La/SSB positivity had higher CXCL13 levels (*p* < 0.0001 and *p* = 0.0003, respectively). The relationship between CXCL13 and anti-Ro/-La positivity survived when total IgG was controlled for. Furthermore, there was a significant association between CXCL13 and ESR (*r* = 0.300, *p* < 0.0001).

**Table 2:** Associations of CXCL13 with serological B-cell and inflammatory markers in primary Sjögren’s syndrome

B-cell/inflammatory marker	Pearson correlation (*r*)	Benjamini–Hochberg adjusted *p* values
BAFF (pg/mL)	0.263	0.0002
B2M (mg/L)	0.459	< 0.0001
CMBY (mg/L)	0.375	< 0.0001
κ light chain (mg/L)	0.358	< 0.0001
λ light chain (mg/L)	0.338	< 0.0001
IgG (mg/mL)	0.320	< 0.0001
κ–λ ratio	0.135	0.0682
IgA (mg/mL)	0.110	0.0996
IgM (mg/mL)	0.084	0.2213
C4 (g/L)	− 0.054	0.4484
C3 (g/L)	− 0.065	0.3531
Anti-Ro/SSA	–	< 0.0001
Anti-La/SSB	–	0.0003
RF	–	0.0039
ESR (mm/1st h)	0.300	< 0.0001
CRP (mg/L)	0.061	0.3581
WCC (× 10^9^/L)	− 0.049	0.4447

Correlations of serological B-cell and inflammatory markers with CXCL13 concentrations measured for pSS patients without NHL at visit 1. The Pearson correlation coefficients (*r*) were calculated by the pairwise correlation method in JMP statistical software. *p* values lower than 0.05 were considered statistically significant. The false discovery rate (FDR) was controlled using the Benjamini–Hochberg (B–H) correction method

*BAFF* B-cell activating factor, *B2M* β2 microglobulin, *CMBY* combined free light chains, *IgG* immunoglobulin G, *IgA* immunoglobulin A, *IgM* immunoglobulin M, *RF* rheumatoid factor, *ESR* erythrocyte sedimentation rate, *CRP* C-reactive protein, *WCC* white blood cell count

### IgG and C3 are NHL risk factors in pSS

While CXCL13 is significantly associated with NHL risk score, this may arise indirectly as a result of its association with the B-cell and inflammatory markers discussed in “CXCL13 is associated with B cell and inflammatory markers”. Accordingly, we modelled NHL risk as a function of CXCL13 and other candidates using logistic regression. There were sixteen candidate variables for inclusion in the model. To construct this model, we first screened these using univariate logistic regression identifying those candidates most closely associated with NHL risk. NHL risk was significantly associated with CXCL13, CMBY, κ light chain, λ light chain, IgG, IgM, B2M, ESR, and C3. All nine candidates were entered into a multiple logistic regression model along with age and sex, and the remaining candidates were eliminated using a backwards stepwise method minimising the AIC criterion. Just two candidates, IgG and C3, survived backward elimination. Higher IgG levels were associated with higher NHL risk (*p* < 0.0001) and lower C3 levels with lower NHL risk (*p* = 0.0041). While the CXCL13 effect was robust to inclusion of either IgG or C3, it was sensitive to the inclusion of both (*p* = 0.0554). We note that CXCL13 is weakly associated with NHL risk, once IgG and C3 levels are included in the model.

## Discussion

This study demonstrates that serum CXCL13 levels are elevated in all pSS groups compared to healthy individuals and are associated with moderate risk for NHL development in pSS. In addition, serum CXCL13 and NHL risk score remain relatively stable over a median 4-year time interval. Serum CXCL13 is also associated with B-cell and inflammatory markers including BAFF, B2M, CMBY, κ and λ light chains, IgG, ESR, RF, Ro/SSA and La/SSB autoantibodies. Moreover, IgG and C3 are associated with NHL risk in pSS.

Several clinical and biological factors have been related with NHL in pSS and used to predict the disease outcome and survival rate of patients [2, 23, 25–27]. Clinical factors include persistent SG enlargement, palpable purpura, fatigue, splenomegaly, lymphadenopathy, peripheral neuropathy, glomerulonephritis and high ESSDAI score [1, 2, 23, 25–28]. Biological risk factors include C4/C3 hypocomplementemia, cryoglobulinaemia, serum monoclonal component, CD4+ T lymphocytopenia, anti-Ro/SSA, anti-La/SSB and RF positivity [2, 23, 25, 28–31]. The formation of ectopic GC in the SG epithelium is another reported risk factor for pSS-associated NHL development [6]. One of the major chemokines involved in the initiation and maintenance of ectopic GCs is CXCL13. Considering this, the purpose of the present study was to investigate the role of serum CXCL13 as a potential NHL risk biomarker in pSS.

In this study, we found increased CXCL13 concentrations in the serum of pSS patients when compared to healthy individuals. Also, pSS patients with a history of NHL had higher serum CXCL13 levels than those without. Similar findings were presented in a previous study that included patients from the ASSESS cohort [19].

Serum CXCL13 levels were found to be higher in both the MR and pSS-NHL+ groups compared to the LR group, but no difference was found between the MR and pSS-NHL+ groups. CXCL13 could not discriminate between the MR and pSS-NHL+ groups but could distinguish the LR from the MR group which may indicate a role of CXCL13 in the early stages of the pathophysiological process of lymphoma development. Rather than supporting the proliferation or survival of malignant B cells in established NHL, CXCL13 may play a role in autoreactive B-cell generation and expansion in ectopic GC predisposing patients to NHL development. Ectopic GC express activation-induced cytidine deaminase (AID) which supports the class switch recombination (CSR) and somatic hypermutation (SHM) of the Ig genes. Deregulation of CSR and SHM can favour the selection of autoreactive GC B-cell clones. It is shown that AID is invariably expressed in the ectopic GC B cells in the pSS minor SG and in parotid pSS mucosa-associated lymphoid tissue (MALT) [32]. The potential role of CXCL13 in autoreactive B-cell generation is described in a different study where immunohistochemistry and RT-PCR analysis results demonstrated increased CXCL13 transcripts in SS minor SGs in the reactive follicular B-cell component [11].

Overall serum CXCL13 levels remained stable between the two visits over a median of a 4-year period. It would be interesting to measure changes in serum CXCL13 levels over a longer duration as a potential biomarker of disease progression. Also, among pSS-nonL patients at visit 1, three developed NHL at visit 2. One of the patients was in the HR group, whereas the other two were in the MR group. None of the LR patients developed NHL within the follow-up period.

Studies have reported that specific B-cell markers such as monoclonal immunoglobulins, free light chains found in the serum and urine, and increased serum BAFF levels are associated with lymphoma in pSS patients [9, 20]. Based on these studies, we might expect a correlation between CXCL13 levels and B-cell markers. We found that CXCL13 levels were positively correlated with IgG. IgG is known to be highly expressed by the SG in pSS and may contribute to the uncontrolled stimulation of RF positive B cells within GC [33]. CXCL13 levels were also positively correlated with BAFF, B2M, CMBY, κ light chain, λ light chain, anti-Ro/SSA, anti-La/SSB and RF. The observed correlation between CXCL13 and these markers may support the role of CXCL13 in lymphoma. However, the exact pathway is unknown and deserves further investigation.

Finally, we modelled NHL risk as a function of CXCL13 and other candidates using logistic regression. The final model included IgG and C3 controlled for age and sex. CXCL3 was weakly associated with NHL risk in the inclusion of both IgG and C3 but its effect was robust to the inclusion of either IgG or C3. Further study is required to examine the relationship and interactions between C3, IgG and CXCL13.

A key strength of our study is the large number of patient participants included from different centres across the UK, increasing its ecological relevance. However, further studies are needed to confirm the findings using longitudinal studies with longer follow-up and in other populations.

There are some limitations to our study. CXCL13 concentrations were measured in the serum, rather than in SGs, from where most NHL arise. However, previous studies have shown that CXCL13 is not only expressed locally, but also it can be found in the serum and plasma of patients [19, 34]. In addition, biopsy is an invasive process and not all patients are willing to provide a biopsy for research or for the diagnostic process. Therefore, serum CXCL13 may be more appealing as biomarker than SG CXCL13. A further limitation is that we cannot exclude the possibility that medications used to treat patients with NHL prior to the blood collection for this study may affect serum CXCL13 concentrations. Another limitation is that Fragkioudaki’s risk model has yet to be validated in an independent study. However, although many clinical and biological factors have been described as lymphoma predictors in pSS, there are no widely accepted models for assessing lymphoma risk. Furthermore, based on the data from this study, the incidence of progression to lymphoma among the LR, MR and HR groups was 0%, 3% and 100%, respectively, giving some support to the validity of Fragkioudaki’s scoring system.

In conclusion, this study has demonstrated that serum CXCL13 levels are associated with NHL risk and occurrence in pSS. Further research to explore serum CXCL13 as a potential NHL risk biomarker in pSS is warranted.

## Electronic supplementary material

Below is the link to the electronic supplementary material.

Supplementary file1 (DOCX 13 kb)

Supplementary file2 (DOCX 44 kb)
