# Supplementary material for: Serum CXCL13 levels are associated with lymphoma risk and lymphoma occurrence in primary Sjögren’s syndrome
Source: Rheumatol Int. 2020 Feb 11;40(4):541–8. doi: 10.1007/s00296-020-04524-5 (PMC7069897; doi:10.1007/s00296-020-04524-5)
Supplement: Supplementary file 1 — Supplementary file1 (DOCX 13 kb) [file 296_2020_4524_MOESM1_ESM.docx]

Table S1 - Lymphoma types in pSS-NHL+ visit 1 and visit 2 patients

| Number of pSS-NHL+ patients | Marginal node lymphoma of MALT type | Diffuse B-cell Lymphoma | NHL | Not specified | Hodgkin Lymphoma |
| --- | --- | --- | --- | --- | --- |
| Visit 1 patients (N) | 23 | 1 | 5 | 6 | 3 |
| Visit 2 patients (N) | 10 | - | 1 | 1 | - |
