# Supplementary material for: Serum CXCL13 levels are associated with lymphoma risk and lymphoma occurrence in primary Sjögren’s syndrome
Source: Rheumatol Int. 2020 Feb 11;40(4):541–8. doi: 10.1007/s00296-020-04524-5 (PMC7069897; doi:10.1007/s00296-020-04524-5)
Supplement: Supplementary file 2 — Supplementary file2 (DOCX 44 kb) [file 296_2020_4524_MOESM2_ESM.docx]

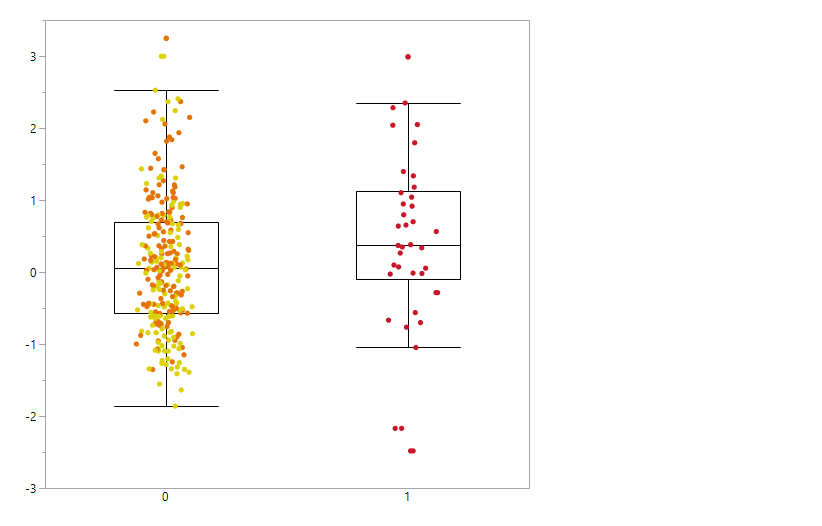


*p* = 0.0204

pSS-nonL pSS-NHL+

Log[CXCL13(pg/mL)]

**Fig. S2** – Comparison of serum CXCL13 levels between pSS-nonL and pSS-NHL+ patients at Visit 1. CXCL13 serum levels were significantly higher in pSS-NHL+ patients than in pSS-nonL patients (p =0.0204). Comparison of serum CXCL13 concentrations was performed using the Wilcoxon Signed Rank test in JMP. The whisker bars show the median CXCL13 concentration and the boxes represent the quartiles Q1 and Q3. Each dot represents an individual patient. The values beyond the bars are outliers that were included in the analysis. Median [IQR] in pg/mL in the pSS-nonL group: 268 [170-454], Median [IQR] in pg/mL in the pSS-NHL+ group: 268 [163-470].
